# CmirC: an integrated database of clustered miRNAs co-localized with copy number variations in cancer

**DOI:** 10.1007/s10142-022-00909-w

**Published:** 2022-10-26

**Authors:** Akshay Pramod Ware, Kapaettu Satyamoorthy, Bobby Paul

**Affiliations:** 1 Department of Bioinformatics, Manipal School of Life Sciences, Manipal Academy of Higher Education, Manipal, 576104 Karnataka India; 2 Department of Cell and Molecular Biology, Manipal School of Life Sciences, Manipal Academy of Higher Education, Manipal, 576104 Karnataka India

**Keywords:** Copy number variations, Clustered miRNAs, miRNA database, Automated pipeline

## Abstract

Genomic rearrangements and copy number variations (CNVs) are the major regulators of clustered microRNAs (miRNAs) expression. Several clustered miRNAs are harbored in and around chromosome fragile sites (CFSs) and cancer-associated genomic hotspots. Aberrant expression of such clusters can lead to oncogenic or tumor suppressor activities. Here, we developed *CmirC* (**C**lustered **miR**NAs co-localized with **C**NVs), a comprehensive database of clustered miRNAs co-localized with CNV regions. The database consists of 481 clustered miRNAs co-localized with CNVs and their expression patterns in 35 cancer types of the TCGA. The portal also provides information on CFSs, miRNA cluster candidates, genomic coordinates, target gene networks, and gene functionality. The web portal is integrated with advanced tools such as JBrowse, NCBI-BLAST, GeneSCF, visNetwork, and NetworkD3 to help the researchers in data analysis, visualization, and browsing. This portal provides a promising avenue for integrated data analytics and offers additional evidence for the complex regulation of clustered miRNAs in cancer. The web portal is freely accessible at http://slsdb.manipal.edu/cmirclust to explore clinically significant miRNAs.

## Introduction

Cancer is one of the major non-communicable diseases with high incidence and mortality rates (Sung et al. 2021). Several factors including genetic and epigenetic alterations participate in carcinogenesis (You and Jones 2012). Despite the architecture of human genome majorly consisting of non-coding regions, a large number of studies have focused on cancer-causing genomic alterations in protein-coding regions (Gloss and Dinger 2018). However, the non-coding genomic regions can drive essential biological functions and control the expression of genes that are involved in several diseases, including cancer. Small non-coding miRNAs regulate gene expression via targeting mRNAs and have been shown to act as oncogenes or tumor suppressors under certain conditions (Peng and Croce 2016; Oh et al. 2017). Amplification or deletion of miRNA genes, their abnormal transcriptional regulation, and defects in the biogenesis pathway can alter the miRNA expression profiles in cancer patients (Peng and Croce 2016).

**Fig. 1:**
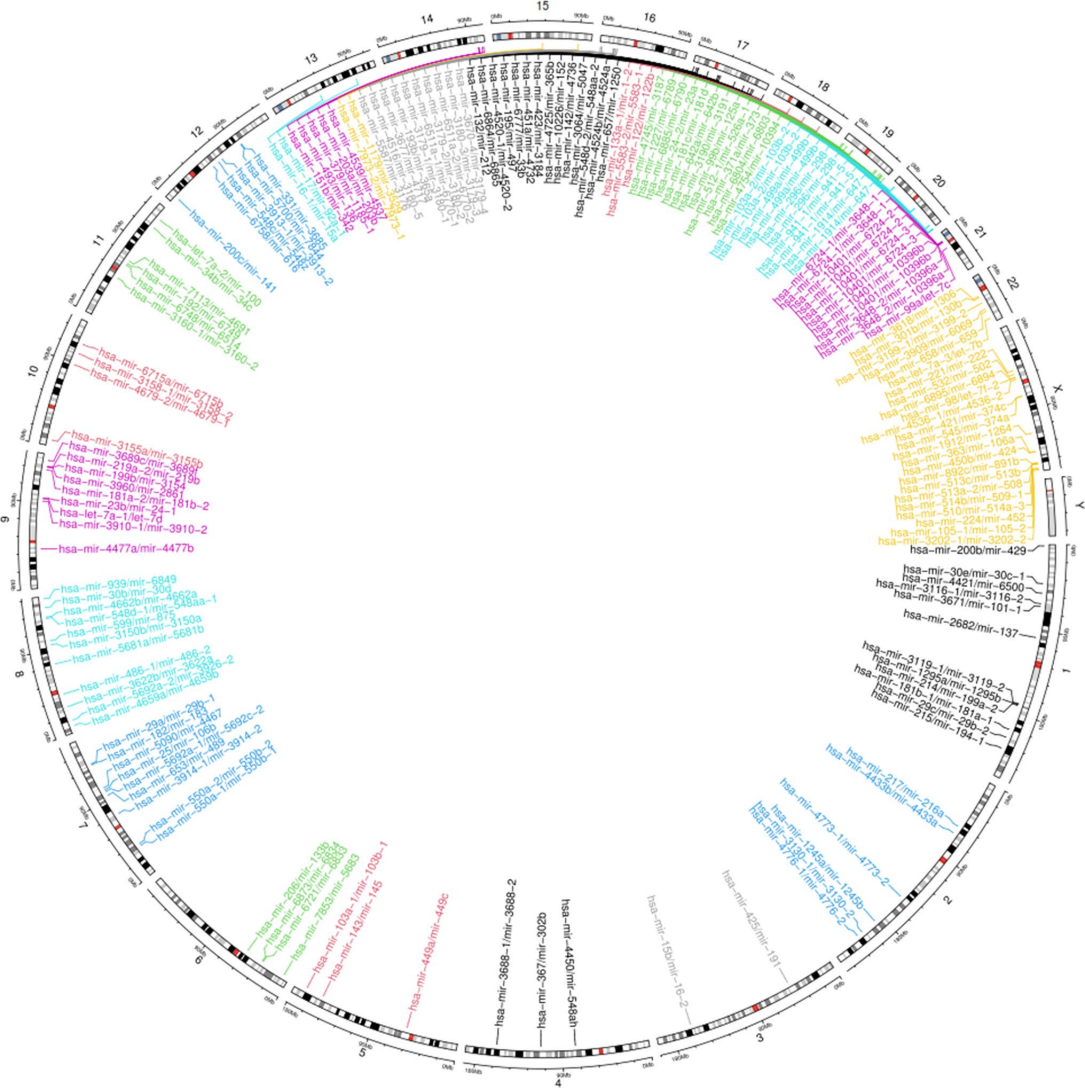
The distribution of 159 miRNA clusters in the human genome. Highest number of miRNA clusters (18) is harbored on the X chromosome

The miRBase offers information on miRNAs and the latest version (v22.1) consists of 2654 mature human miRNAs (Kozomara et al. 2019). Figure 1 illustrates the 481 miRNAs belonging to 159 clusters spanning all the chromosomes. Each cluster consists of more than one miRNA transcribed from physically adjacent positions driven by a single promoter region (Seitz et al. 2004). The cluster members show high sequence similarity in the seed region, and they often target the same or different genes belonging to a specific pathway. Hence, the effect due to the abnormal expression of clustered miRNAs could be more severe than non-clustered miRNAs.

Aberrantly expressed clusters are regulated by genetic and epigenetic mechanisms such as mutations, deletions, amplifications, and DNA methylation (Kabekkodu et al. 2018). These in turn can alter the mRNA translation, signal transduction pathways, metabolic flux, or protein function. Dysregulated expression of CNV-driven clustered miRNAs in carcinomas of the ovary (Zhang et al. 2008), gastrointestinal tract (An et al. 2013), lung (Xia et al. 2018), and urinary bladder (Ware et al. 2022) has been well documented. However, the interplay between clustered miRNAs and CNVs in all cancer types is largely unknown. Nearly 50% of the miRNA genes are co-localized with the CFSs that exhibit higher genomic instability and frequent copy number changes. Furthermore, they also affect the expression of clustered miRNAs (Calin et al. 2004; Sevignani et al. 2007; Kabekkodu et al. 2018).

In recent years, numerous databases such as miRCancer (Xie et al. 2013), miRwayDB (Das et al. 2018), TACCO (Chou et al. 2019), miRactDB (Tan et al. 2021), DriverDBv3 (Liu et al. 2020), and miR-TV (Pan and Lin 2020) have been released for public access to comprehend cancer drivers. Additionally, well established computational approaches such as miRDriver (Bose and Bozdag 2019), CAMIRADA (Shamsizadeh et al. 2019), FCMDAP (Li et al. 2019), and PMAMCA (Ha et al. 2019) can determine the associations between microRNAs and cancers. However, a comprehensive resource for miRNA clusters co-localized with CNV regions and their expression analysis is unavailable. Hence, it is necessary to integrate genetic and epigenetic data associated with the miRNA clusters, for the critical evaluation of cancer progression and the development of therapeutics.

The present study integrated multi-omics datasets from the 35 TCGA cancer types and developed a user-friendly database, **C**lustered **miR**NAs co-localized with **C**NVs (*CmirC*). With advanced search and browse options, the portal allows users to explore and analyze the datasets of individual clustered miRNAs co-localized with CNVs, CFSs, and their regulation in different cancer types.

## Materials and methods

**Fig. 2:**
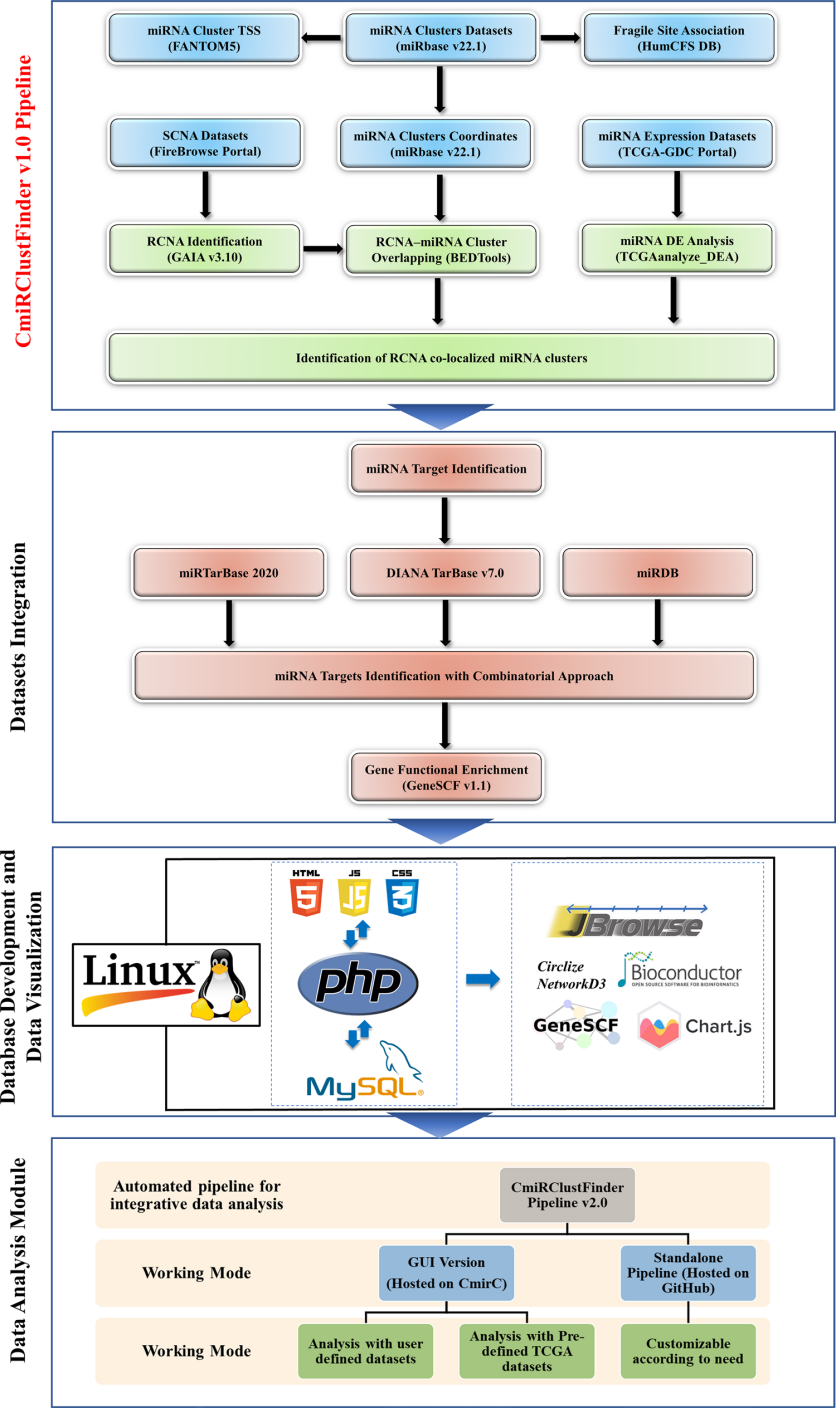
Schematic representation of data collections, analysis, integration, and *CmirC* database development. All the data is referenced against the human reference genome build hg38

The study consists of two major parts: (i) integration of CNV—clustered miRNA data analysis of 35 cancer types, and (ii) development of a database for clustered miRNAs co-localized with CNVs. We have used publicly available CNV, clustered miRNA, RNASeq datasets, and bioinformatics resources for integrated data analysis. Computer languages such as hypertext markup language (HTML), PHP: hypertext pre-processor (PHP), JavaScript, and MySQL were used to develop this interactive database. The schematic representation of data collection, analysis, integration, and *CmirC* database development is illustrated in Fig. 2.

### Data collection and sources

Currently, 159 miRNA clusters are reported from the human genome and each cluster consists of two or more miRNAs. Primary information on 159 miRNA clusters consisting of 481 precursors and 717 mature miRNAs was retrieved from the miRBase v22.1 database (http://www.mirbase.org/). The transcription start sites (TSS) for clustered miRNAs were retrieved from the FANTOM5 repository (Lizio et al. 2019) and the CFSs data was downloaded from the HumCFS database (Kumar et al. 2019). Pre-computed segmented copy number aberrations CNA (SCNA) data for 35 cancer types were obtained from the Broad Institute’s FireBrowse portal (http://firebrowse.org/). Level 3 miRNA expression datasets from the TCGA-GDC portal (https://portal.gdc.cancer.gov/) were interrogated using the R package TCGAbiolinks (Colaprico et al. 2016). Potential miRNA target genes were identified and retrieved from miRTarBase (Huang et al. 2020), DIANA-TarBase (Vlachos et al. 2015), and miRDB (Chen and Wang 2020) repositories. Only those target gene(s) for miRNAs that are reported by these three databases were considered for further downstream analysis.

### Integrated data analysis

Recurrent CNVs (RCNVs) in the samples of 35 cancer types were analyzed using GAIA 3.10, R Bioconductor package. The probe metadata file at the Broad Institute’s data portal (ftp://ftp.broadinstitute.org/pub/GISTIC2.0/hg19_support/) was used to obtain information on RCNV cytoband and their location. The RCNVs are defined by false discovery rate (FDR) *Q* score < 0.15 from 10 iterations. The segmental mean of 0.3 was set as the threshold to identify the copy number gain or loss. The regions with a mean threshold value > 0.3 and ≤ 0.3 were considered as a copy number gain (amplification) and loss (deletion), respectively. UCSC LiftOver (https://genome.ucsc.edu/cgi-bin/hgLiftOver), a genome upgradation tool, was used to lift all SCNA genomic coordinates to match with the hg38 genome build. Further, BEDTools (Quinlan and Hall 2010) were used to intersect the genomic coordinates of miRNA clusters onto the recurrent significant CNV regions. The intersect function of the BEDTools was used to map coordinates of 159 miRNA cluster regions on fragile sites. The CRAN package “circlize” was used for the graphical representation of significant CNV and miRNA cluster co-localization (Gu et al. 2014). A quantile filtration with a cut-off value of 0.25 was used to filter the significant miRNAs and thereby excluding the miRNAs with very low read count. For the differential expression (DE) analysis, we used the TCGAanalyze_DEA package with various functions of the edgeR package from Bioconductor (Robinson et al. 2010). The function “glmLRT” was employed to make pair-wise tests and DE analysis between the two groups. The *p* values obtained were sorted in ascending order and further adjusted using the FDR correction to shortlist the top differentially expressed miRNAs. Thresholds for logarithmic fold change (Log2FC) and FDR were set as 1 and 0.1 respectively, such that differentially expressed miRNAs were considered to be significant only if Log2FC > 1 and FDR < 0.05.

### Database construction, web interface, and data visualization

The LAMP, a Linux-based stack of open-source software consisting of Apache v2.4, MySQL v5.7, and PHP v7.2, was used for the development of *CmirC*. The database is hosted on the server with the Red Hat Enterprise release 6.10 operating system. Data has been categorized and stored in tabular format using MySQL relational database management system for efficient administration and management. The interactive user interface was built using HTML, cascading style sheet, bootstrap, and JavaScript. The intermediate layer and server-side scripting were performed using PHP. An interactive genome browser was configured using JBrowse (Skinner et al. 2009) to provide fast and smooth scrolling of identified RCNA from 35 cancers and co-localized miRNA clusters. The NCBI BLAST + v2.5.0 (Altschul et al. 1990) has been integrated into the *CmirC* to provide sequence similarity-based searching. We incorporated two R packages, visNetwork (https://github.com/datastorm-open/visNetwork), and networkD3 (https://github.com/christophergandrud/networkD3) to portray the miRNA-target gene interaction networks. Command-line tool gene set clustering based on functional annotation (GeneSCF) v1.1 (Subhash and Kanduri 2016) has been configured on the *CmirC* server allowing the user to perform the enrichment analysis of a set of miRNA target genes. The expression profiles of miRNAs are presented as a bar graph using additional JavaScript packages, Chart.js (https://www.chartjs.org/), and CanvasJS (http://canvasjs.com/).

## Results

### Integrated data analysis

**Fig. 3:**
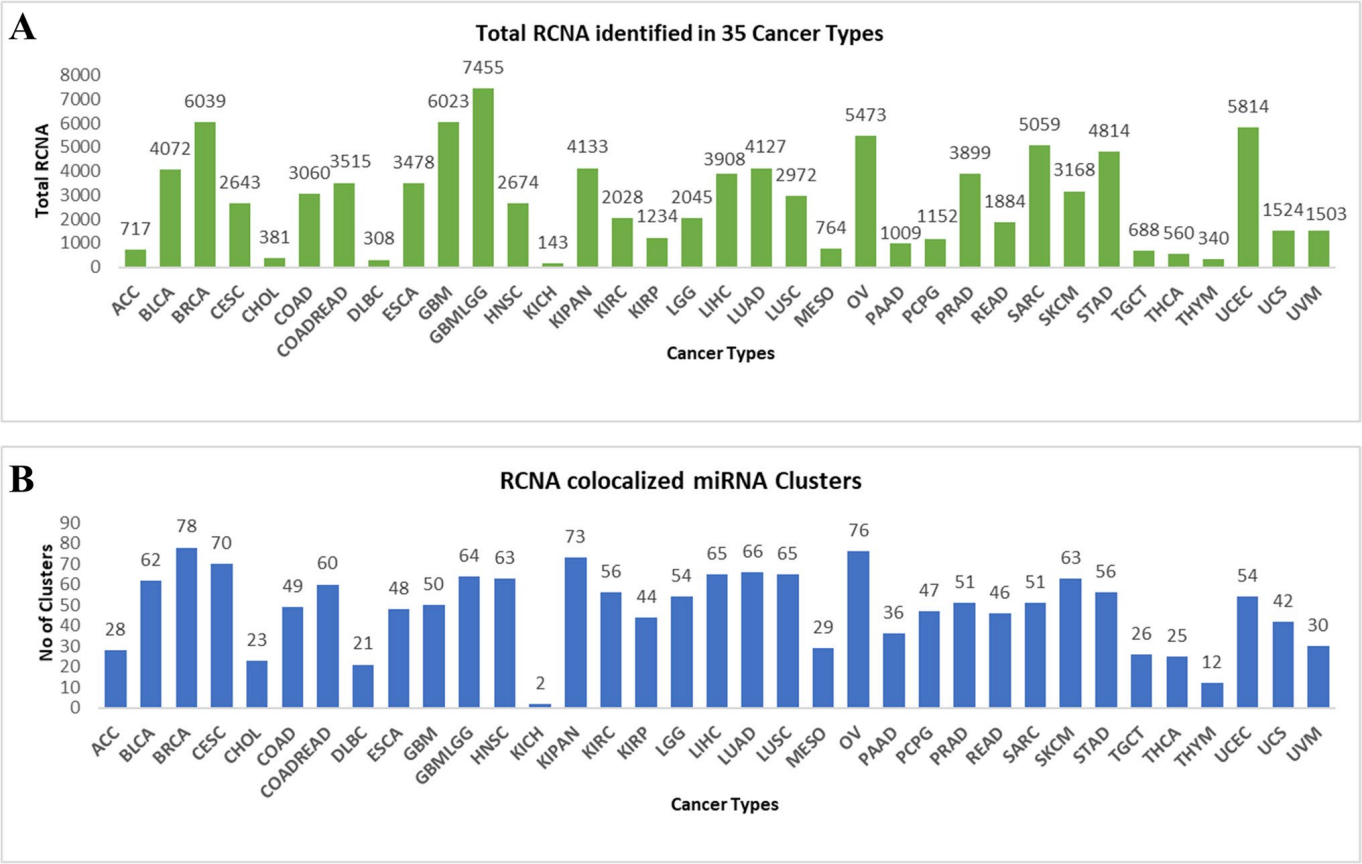
Data statistics of the *CmirC* portal. **A** The significant RCNA was identified across the 35 cancer types. **B** Total number of miRNA clusters co-localized with RCNA in each cancer type

The *CmirC* is an open-access platform that provides information on miRNA clusters and their co-localization with RCNVs from the TCGA cancer types. A total of 12,496 CNVs and 10,461 miRNA expression samples were downloaded from the TCGA database for the integrated data analysis and database development. We have identified 125 miRNA clusters co-localized with RCNVs across 35 cancer types. The top 10 cancer types which were more prone to genomic instability events are glioma (GBMLGG), breast invasive carcinoma (BRCA), glioblastoma multiforme (GBM), uterine corpus endometrial carcinoma (UCEC), ovarian cancer (OV), sarcoma (SARC), stomach adenocarcinoma (STAD), lung adenocarcinoma (LUAD), urothelial carcinoma (BLCA), and hepatocellular carcinoma (LIHC). A total of 48,876 RCNVs events were identified in these cancers. The highest number of miRNA clusters were found to be co-localized with RCNA in BRCA (78) followed by OV (70), GBMLGG (64), LUAD (66), and BLCA (62). The total number of identified RCNVs and co-localized miRNA clusters from the individual cancer types is illustrated in Fig. 3. Interestingly, we identified co-localization of hsa-mir-199a/mir-214 and hsa-mir-657/mir-1250 miRNA clusters with amplified region in 28 and 21 cancer types, respectively. Similarly, hsa-mir-3926–2/mir-5692a-2 cluster was found in deleted region of 29 cancer types. The RCNV and miRNA cluster relation is depicted as an interactive network and is provided in the information section of *CmirC* portal.

**Fig. 4:**
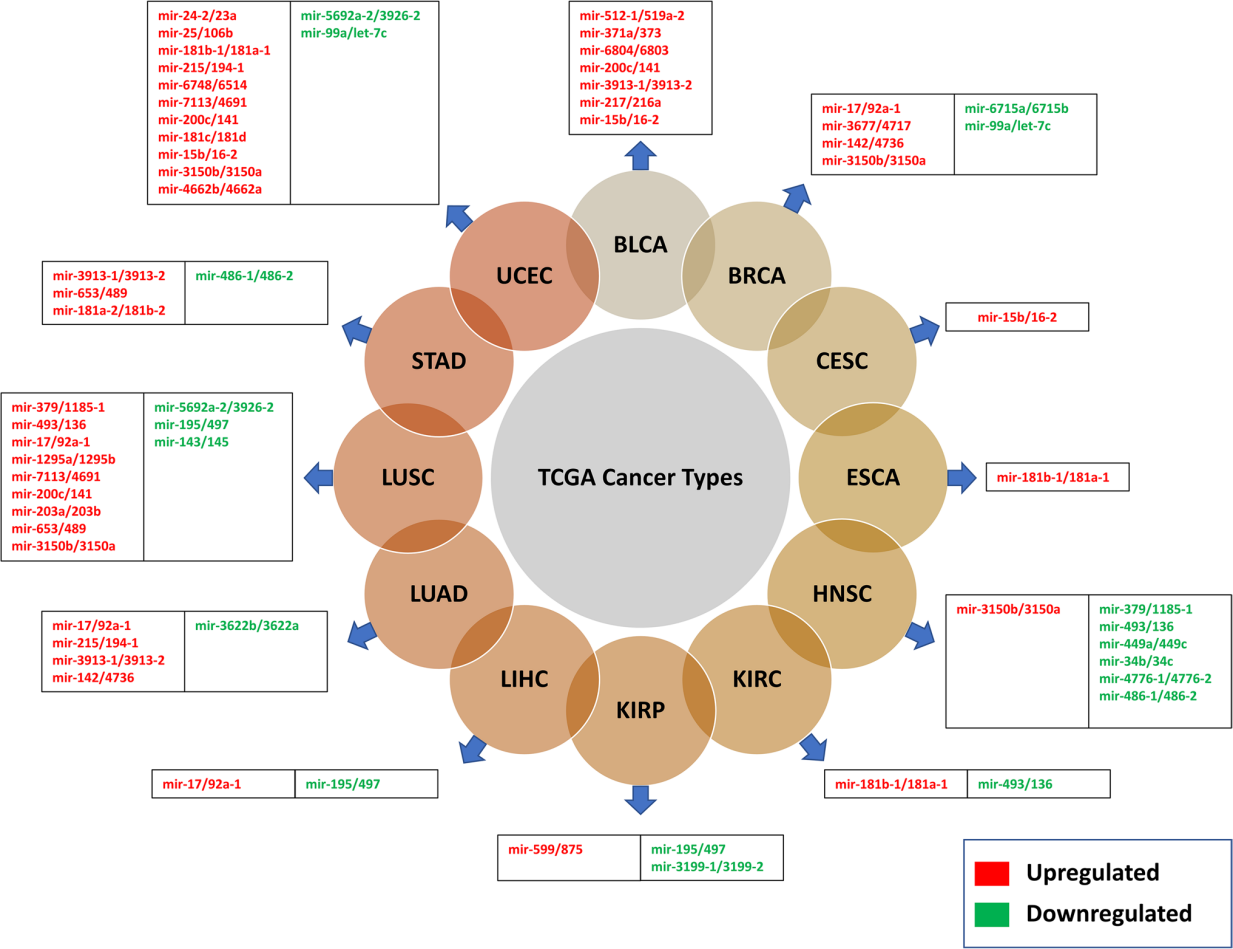
The CNV-driven differentially expressed miRNA clusters (normal vs tumor) across 12 cancer types

A large proportion of the genetic and epigenetic reprogramming influences the miRNA promoter and transcription start sites (TSS). Considering this, we identified potential TSS for 149 miRNA clusters from the FANTOM5 repository. Also, by intersecting miRNA cluster coordinates on fragile sites, we identified 57 miRNA clusters spanning 29 fragile sites on different chromosomes (Table 1). Out of these 29, the frequency of 19 fragile sites is common, nine are rare, and one is unknown. The chromosome 19 fragile sites (FRA19A and FRA19B) have the highest miRNA cluster co-localization (7 and 5, respectively), and the largest miRNA cluster hsa-mir-512–1/mir-1283–1 (C19MC) is co-localized with FRA19A. The integrated RCNA and cluster candidate’s differential expression showed that 46 miRNA clusters associated with 12 cancer types were significantly correlated. Of which, the expression of 32 miRNA clusters was upregulated, whereas 14 clusters were downregulated (Fig. 4).

**Table 1:** Chromosome-wise distribution of fragile sites and associated miRNA clusters

Fragile site	Position	Length (bp)	Classification	miRNA cluster(s)
FRA1A	Chr1:1–28,000,000	28,000,000	Common	hsa-mir-429/mir-200b
FRA1B	Chr1:50,700,001–61,300,000	10,600,000	Common	hsa-mir-6500/mir-4421
FRA1L	Chr1:61,300,001–84,900,000	23,600,000	Common	hsa-mir-3116–2/mir-3116–1, hsa-mir-101–1/mir-3671
FRA1M	Chr1:94,700,001–99,700,000	5,000,000	Rare	hsa-mir-137/mir-2682
FRA10D	Chr10:74,900,000–1,070,600,001	995,700,002	Common	hsa-mir-4679–1/mir-4679–2, hsa-mir-3158–2/mir-3158–1, hsa-mir-6715b/mir-6715a
FRA11EH	Chr11:63,400,001–77,100,000	13,700,000	Common	hsa-mir-6749/mir-192, hsa-mir-4691/mir-7113
FRA16A	Chr16:14,800,001–16,800,000	2,000,000	Rare	hsa-mir-3180–1/mir-3179–1, hsa-mir-3180–2/mir-3179–2, hsa-mir-6770–1/mir-6511a-1, hsa-mir-6770–2/mir-6511a-2
FRA18B	Chr18:53,800,001–61,600,000	7,800,000	Common	hsa-mir-122b/mir-122
FRA19A	Chr19:32,400,001–59,128,983	26,728,983	Common	hsa-mir-526a-1/mir-512–1, hsa-mir-373/mir-371a, hsa-mir-125a/mir-99b, hsa-mir-6803/mir-6804, hsa-mir-642b/mir-642a, hsa-mir-3191/mir-3190, hsa-mir-10394/mir-4754
FRA19B	Chr19:1–20,000,000	20,000,000	Rare	hsa-mir-23a/mir-24–2, hsa-mir-3187/mir-4745, hsa-mir-6789/mir-1227, hsa-mir-6790/mir-6885, hsa-mir-181d/mir-181c
FRA2S	Chr2:44,100,001–154,900,000	110,800,000	Common	hsa-mir-216a/mir-217, hsa-mir-4433a/mir-4433b, hsa-mir-4773–2/mir-4773–1
FRA2I	Chr2:197,400,001–209,000,000	11,600,000	Common	hsa-mir-3130–2/mir-3130–1
FRA3D	Chr3:148,900,001–160,700,000	11,800,000	Common	hsa-mir-16–2/mir-15b
FRA5G	Chr5:168,500,001–180,915,260	12,415,260	Rare	hsa-mir-103b-1/mir-103a-1
FRA6B	Chr6:4,200,001–7,100,000	2,900,000	Common	hsa-mir-5683/mir-7853
FRA6H	Chr6:30,400,001–46,200,000	15,800,000	Common	hsa-mir-6833/mir-6721, hsa-mir-6834/mir-6873
FRA7F	Chr7:98,000,001–107,400,000	9,400,000	Common	hsa-mir-106b/ mir-25, hsa-mir-4467/mir-5090
FRA7J	Chr7:59,900,001–77,500,000	17,600,000	Common	hsa-mir-3914–2/mir-3914–1
FRA7H	Chr7:130,400,001–132,600,000	2,200,000	Common	hsa-mir-29b-1/mir-29a
FRA8D	Chr8:139,900,001–146,364,022	6,464,022	Common	hsa-mir-6849/mir-939
FRA8B	Chr8:93,300,001–99,000,000	5,700,000	Common	hsa-mir-3150a/mir-3150b
FRA8C	Chr8:117,700,001–127,300,000	9,600,000	Common	hsa-mir-548aa-1/mir-548d-1, hsa-mir-4662a/mir-4662b
FRA9D	Chr9:90,400,001–91,800,000	1,400,000	Common	hsa-mir-3910–2/mir-3910–1
FRAXA	ChrX:141,900,001,147,100,000	5,200,000	Rare	hsa-mir-891b/mir-892c
FRAXE	ChrX:147,100,001–155,270,560	8,170,560	Rare	hsa-mir-508/mir-513a-2, hsa-mir-509–1/mir-514b, hsa-mir-514a-3/mir-510, hsa-mir-105–2/mir-105–1, hsa-mir-513b/mir-513c, hsa-mir-452/mir-224, hsa-mir-3202–2/mir-3202–1
FRA10A	Chr10:97,000,000–1,089,500,001	992,500,002	Rare	hsa-mir-365b/mir-4725, hsa-mir-152/mir-10226
FRA8E	Chr8:117,700,001–127,300,000	9,600,000	Rare	hsa-mir-502/mir-532, hsa-mir-106a/mir-363
FRA10AC1	Chr10:101,900,000–1,099,300,001	997,400,002	–	hsa-mir-365b/mir-4725
FRA10B	Chr10:111,900,001–114,900,000	3,000,000	Rare	hsa-mir-152/mir-10226

### The *CmirC* interface

**Fig. 5:**
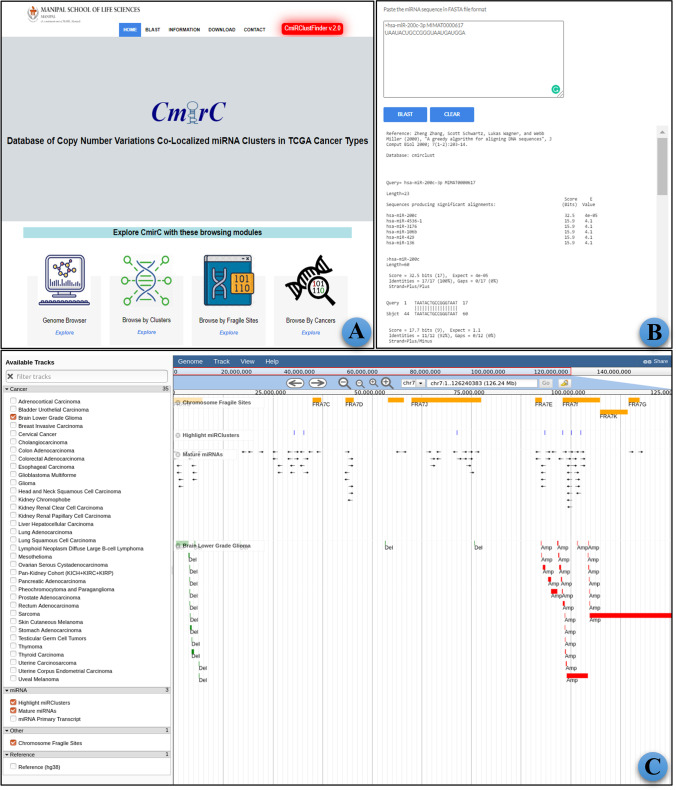
The graphical user interface of *CmirC* web server for the browsing and analytics. **A** Homepage with various browse options. **B** Interactive genome browser page. **C** The webpage for BLAST-based sequence similarity search

The homepage of the *CmirC* web portal provides a quick browse option, wherein users can explore the repository by selecting specific miRNA clusters, fragile sites, or cancer types (Fig. 5A). All these browsing options are indexed in a tabular form for easy and efficient access. We have provided an option to perform a BLAST-based sequence similarity search against clustered miRNA sequences at the *CmirC*. This facilitates fast and accurate identification of clustered miRNAs from user-given datasets. The help page provides a user manual for easy navigation of resources provided in the *CmirC*. The datasets can be downloaded in various file formats such as tab-delimited, comma-separated, XLSX files, PDF, HTML, BED, PNG, and JPEG for further downstream data analysis or presentation.

### Key features and utilities of *CmirC*

#### Browse options

The *CmirC* provides multiple straightforward browsing facilities to access the integrated datasets. Users can browse the database in three ways: (i) by miRNA cluster name that provides information about 159 miRNA clusters, including genomic coordinates, cluster candidates, and CNV information; (ii) by fragile sites that lists all the miRNA clusters on the chromosome fragile sites with their genomic location information; and (iii) by cancer type. Users can retrieve RCNAs and their co-localized miRNA clusters by selecting the specific cancer type. Further, the hyperlink has been enabled to fetch detailed information on individual miRNA clusters.

#### Sequence alignment

The stable version of NCBI BLAST + (v2.5.0) is configured to perform a sequence similarity search (Fig. 5B). The server executes the BLASTN alignment algorithm for the user-uploaded sequence against the clustered miRNA sequences. This option allows the identification of clustered miRNAs homologous to the query sequence.

#### Genome browser

The *CmirC* is integrated with an interactive tool JBrowse to visualize miRNA cluster co-localization in the RCNV regions (Fig. 5C). This genome browser allows users to quickly view CNV regions, fragile sites, and their co-localized miRNA clusters with extended zoom levels for higher resolution. The display includes multiple parallel tracks of annotated features such as reference sequences, precursor and mature miRNAs, fragile sites, and RCNA from each cancer type. This feature facilitates cumulative visualization and seamless navigation between the tracks. Users can conveniently navigate through miRNA clusters, CNV regions, and CFSs. Details about mature, precursor as well as clustered miRNAs, CNV areas, and CFSs in the genome (hg38 build) appear in a pop-up window. Using the highly flexible and customizable option of JBrowse, users can easily upload their data, analyze, and download the reports.

#### CNV plots and miRNA-gene networks

**Fig. 6:**
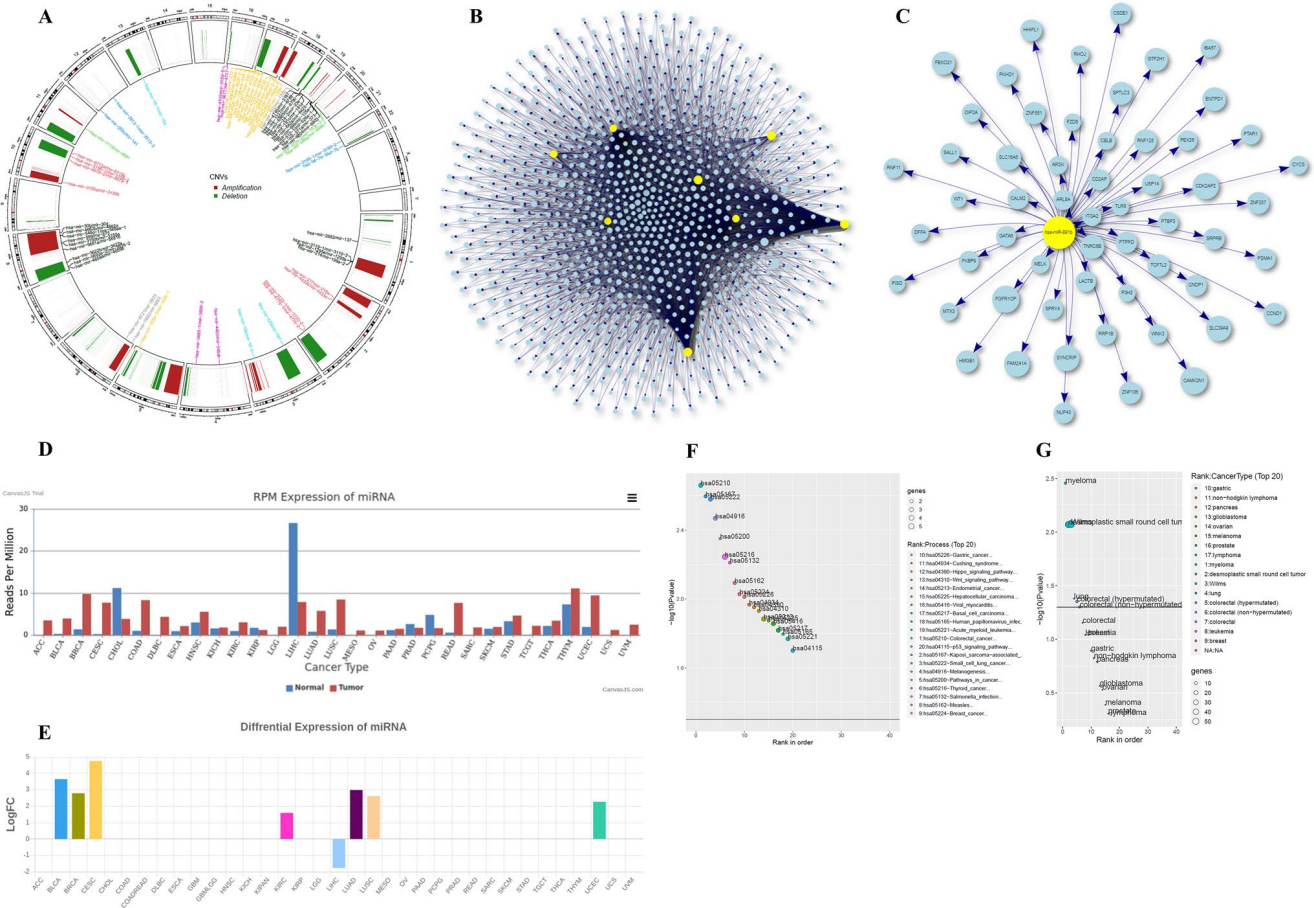
The integrated data representation is provided by the *CmirC*. **A** Circos plot illustrating identified RCNAs and co-localized miRNA Clusters. **B** MiRNA cluster and target gene network. **C** Individual miRNA targets. **D** Average RPM expression of miRNAs. **E** The miRNA differential expression (normal vs tumor). **F** Top 20 enriched pathways for gene set targeted by miRNA clusters. **G** Gene set enriched in the cancer network

This comprehensive resource will provide an opportunity to generate high-quality publication-ready graphs and plots for scientific reporting. Users can generate circos plots of integrated data for individual cancer types (Fig. 6A). The web server is implemented with the open-source R packages; visNetwork and networkD3 for the visualization of clustered miRNA and their target genes. These packages provide an easy way for viewing and adjusting the interaction networks (Fig. 6B, C). The visNetwork generated miRNA-target genes interactions provide a dropdown list for each node in the network. Further, the interconnected subnetworks can be viewed upon the node selection from the dropdown list. The users can directly select and tweak individual nodes or edges of interest. The miRNA and target gene list can be downloaded in tab-delimited file format for further analysis.

#### Data analysis module

*CmiRC*lustFinder v2.0 data analysis tool is an upgraded version with GUI of our prior developed pipeline available on GitHub (https://github.com/msls-bioinfo/CmiRClustFinder_v1.0). This program enables users to identify RCNVs in cancer samples, as well as perform co-localization analyses between RCNVs and user-specified genomic areas of interest. We have provided three modules: (i) data analysis portal, in which the user can upload SCNA datasets and gene coordinates in BED format; (ii) analysis with the TCGA cancer types, the user can pick any one of the 35 TCGA cancer type and run co-localization analysis; and (iii) the standalone version that allows user to download and execute the pipeline on Linux operating systems. The pipeline will generate co-localization report in “.tsv” file and circos-based plots as images. This web tool is available for public use on the *CmirC* portal.

### Miscellaneous features

The web portal is automated for data analysis and generates reports in various graphical representations. The publication-ready clustered miRNA expression profile of cancer types can be visualized in bar graphs (Fig. 6D). The differential expression of miRNAs across tumors and control samples of 35 cancer types is provided (displayed in Fig. 6E). In-house, shell scripts embedded with the webserver allow for gene set functional enrichment analysis on target genes in a convenient manner. The complete list of molecular functions (MF), biological processes (BP), cellular components (CC), and network of cancer genes (NCG) can be retrieved in “.tsv” file format. The significance of the analysis can be predicted based on the FDR and the *p* value calculated using Fisher’s exact test. Further, a bubble plot with the top 20 enriched functions (ranked based on log-transformed *p* value) of miRNA targeted genes is also provided (Fig. 6F, G).

In a move towards automated web portals for big data analysis, we have developed a specific database for clustered miRNAs co-localized with CNV regions reported from 35 cancer types. The comprehensive resource embedded with data analytics and visualization packages provides a better choice for researchers to comprehend the clustered miRNA regulation during carcinogenesis.

## Discussion

Understanding primary mechanisms underlying carcinogenesis requires comprehensive annotation of the integrated cancer genetic data. A major portion of the human genome represents non-coding regions. However, these regions harbor functional elements that can regulate the expression of protein-coding genes (Gloss and Dinger 2018). Genetic and epigenetic reprogramming can also influence the transcription of miRNA expression under various physiological conditions (Gulyaeva and Kushlinskiy 2016). Recent evidence suggests that the alteration of protein-coding genes alone cannot constitute the entire molecular basis of tumor development (Xue and He 2014). Also, the vast majority of somatic alterations of the cancer genome are reported in non-coding regions (Cuykendall et al. 2017). The miRNAs are an important class of non-coding genetic elements that regulate gene expression and control multiple biological events (Oh et al. 2017). Studies on altered expressions of individual miRNAs in carcinogenesis are increasingly recognized and explored over the past few decades (Peng and Croce 2016). Besides, a group of miRNAs is found as a cluster at various genomic loci. The proportion of clustered miRNAs is different across the species, and they tend to be evolutionarily conserved. Researchers have proposed that clustered miRNAs can act more efficiently than a single miRNA, as a cluster contains multiple miRNAs (Wang et al. 2016). Clustered miRNAs deregulation is more potent and crucial in cancer signaling pathways and is further responsible for clinical complications such as resistance to therapy (Lin et al. 2020; Becker et al. 2012). Hence, the role of clustered miRNAs as a biomarker for diagnosis, prognosis, treatment, and improved patient care remains to be fully exploited. The genetic and epigenetic reprogramming that alters the cluster miRNA targeted gene regulation is even more complex during carcinogenesis. Considering all these concerns, we have performed an integrated analysis using publicly available cancer datasets from the TCGA.

Identification of RCNV across TCGA cancer types exhibited the top 10 genomic instability-prone cancers. It has been suggested that strong clustered miRNA articulation happens during carcinogenesis, recommending that the profiling of these miRNA groups could be utilized for the clinical diagnosis of cancer (Kabekkodu et al. 2018). By mapping the miRNA clusters on recurrent CNV regions, we have found the maximum number of miRNA clusters co-localized with genomic susceptibility loci in BRCA, OV, GBMLGG, LUAD, and BLCA. Few reports have already indicated an abnormal expression of clustered miRNAs that may contribute to cancer hallmarks acquisition in the above-mentioned tumor types (Molina-Pinelo et al. 2014; Enokida et al. 2016; Yoshida et al. 2021). Interestingly, we observed that specific genomic regions consisting of miRNA clusters are involved in deletions and amplification events depending on the cancer types. However, members of these clusters can behave either as oncogene or as tumor suppressors, depending on the alteration, cell type, or transcriptional events. In recent years, CFSs have been acknowledged as a significant aspect of cancer biology, as these are the regions where most cancer-related genes occur (Kumar et al. 2019). The genetic instability at CFSs leads to dysregulation of the expression of oncogenes or tumor suppressor genes. A thorough understanding of the relationship between CFS-co-localized miRNA clusters and carcinogenesis is needed before therapeutic strategies based on genomic profiles can be determined. Here, we report that ~ 35% of the miRNA clusters are co-localized with 29 CFSs on various chromosomes. The miRNA differential expression profiles (normal vs tumor samples) were correlated with RCNV co-localized clustered miRNAs. A total of 11 CNV-driven downregulated miRNA clusters were identified from UCEC, followed by LUSC (9 clusters) and BLCA (7 clusters). However, six CNV-driven miRNA clusters have been identified as upregulated in HNSC. A total of 12 distinct cancer types associated miRNA clusters induced by CNVs and their expression patterns are shown in Fig. 4. Ultimately, this analysis suggests new insights into the multi-layered complex regulation of clustered miRNAs during tumorigenesis.

Currently, studies on the miRNA cluster regulation during the tumor development are in its infancy. Moreover, there are no comprehensive resources that provide information on the structural variation and functional regulation of miRNA clusters during carcinogenesis. In this regard, we have developed a web portal *CmirC* that provides integrated information on clustered miRNA-CNV co-localization and expression profile of 481 clustered miRNAs in 35 cancer types. The current version of *CmirC* integrates data on CNV, clustered miRNAs, fragile sites, miRNA expression, and their targets as well as multiple bioinformatics tools for convenient data retrieval and analysis. The *CmirC* offers interactive networks of miRNA clusters and their target genes with a gene set functional enrichment facility. All the identified RCNA-miRNA cluster co-localization datasets are provided for downloading along with circos-based graphical representation. A customizable genome browser displays an integrated genetic dataset in individual tracks for quick access. Further, hyperlinks are enabled for browsing all the precursor and mature miRNAs from their parent repository miRBase.

The *CmirC* platform is equipped with multiple genetic and epigenetic effectors that could potentially impact miRNA cluster regulation during carcinogenesis. The integrative high throughput genetic data provided in this resource will also be helpful to understand the important characteristic of tumor heterogeneity. We suggest that alterations identified co-localizing with cluster miRNAs must be systematically and functionally tested to investigate their effects during tumorigenesis. The combined impact of multiple events on clustered miRNAs can be utilized to advance cancer therapeutics and prevention. Also, manipulation of the clustered miRNA expression in a tissue-specific manner can be achieved, and early results are promising for cancer diagnosis and prognosis. We believe that this repository is a valuable resource in cancer biology that can be exploited to assess clinically significant cancer biomarker data.

## Conclusion

The CNV-miRNA integrated study analyzed 12,496 CNVs and 10,461 miRNAs from the TCGA data to understand the cancer type-specific expression. We developed *CmirC*, an online portal to analyze and retrieve multi-omics clustered miRNA associated data from 35 TCGA cancer types. The *CmirC* database and data analysis platform could pave the way for the possible understanding of challenges in dysregulated clustered miRNA-mediated cancers. This functional genomics approach integrates clustered miRNAs, cancer associated CNVs, miRNA targeted genes, and expression datasets in a visualized and interactive manner. We anticipate that *CmirC* will provide helpful information on miRNA clusters’ structural variation and functional regulation during carcinogenesis. Also, the portal for clustered miRNAs co-localized with RCNV regions can play a potential role in the development of biomarkers for the diagnosis and prognosis of various cancers.

## Data Availability

The data stored in CmirC can be freely retrieved, visualized, and downloaded from the portal http://slsdb.manipal.edu/cmirclust/.
